# Interkingdom microbial consortia mechanisms to guide biotechnological applications

**DOI:** 10.1111/1751-7915.13300

**Published:** 2018-07-16

**Authors:** Shu Zhang, Nancy Merino, Akihiro Okamoto, Phillip Gedalanga

**Affiliations:** ^1^ Global Research Center for Environment and Energy based on Nanomaterials Science National Institute for Material Science 1‐1 Namiki Tsukuba Ibaraki Japan; ^2^ Department of Molecular Microbiology and Immunology Norris Comprehensive Cancer Center University of Southern California 1441 Eastlake Street Los Angeles CA 90033 USA; ^3^ Earth‐Life Science Institute Tokyo Institute of Technology, 2‐12‐1‐I7E‐323 Ookayama, Meguro‐ku Tokyo 152‐8550 Japan; ^4^ Department of Earth Sciences University of Southern California, 835 Bloom Walk, SHS 562 Los Angeles CA 90089‐0740 USA; ^5^ Department of Health Science California State University Fullerton, 800 North State College Boulevard Fullerton CA 92831‐3599 USA; ^6^ Present address: Section of Infection and Immunity Herman Ostrow School of Dentistry University of Southern California CA 90089‐0641 USA

## Abstract

Microbial consortia are capable of surviving diverse conditions through the formation of synergistic population‐level structures, such as stromatolites, microbial mats and biofilms. Biotechnological applications are poised to capitalize on these unique interactions. However, current artificial co‐cultures constructed for societal benefits, including biosynthesis, agriculture and bioremediation, face many challenges to perform as well as natural consortia. Interkingdom microbial consortia tend to be more robust and have higher productivity compared with monocultures and intrakingdom consortia, but the control and design of these diverse artificial consortia have received limited attention. Further, feasible research techniques and instrumentation for comprehensive mechanistic insights have only recently been established for interkingdom microbial communities. Here, we review these recent advances in technology and our current understanding of microbial interaction mechanisms involved in sustaining or developing interkingdom consortia for biotechnological applications. Some of the interactions among members from different kingdoms follow similar mechanisms observed for intrakingdom microbial consortia. However, unique interactions in interkingdom consortia, including endosymbiosis or interkingdom‐specific cell–cell interactions, provide improved mitigation to external stresses and inhibitory compounds. Furthermore, antagonistic interactions among interkingdom species can promote fitness, diversification and adaptation, along with the production of beneficial metabolites and enzymes for society. Lastly, we shed light on future research directions to develop study methods at the level of metabolites, genes and meta‐omics. These potential research methods could lead to the control and utilization of highly diverse microbial communities.

## Importance of interkingdom microbial consortia

The utilization of microbial consortia composed primarily of bacteria to obtain desirable features has increased over the past few decades (Brenner *et al*., 2007; Großkopf and Soyer, 2014; Berlanga and Guerrero, 2016; Dang and Lovell, 2016). Recent technological advancements have revealed key interactions within microbial consortia which has led to the improved application and design for biotechnological processes in natural and engineered systems. However, the scope of applications for intrakingdom consortia has its limits due to, for example, a lack of organelles, genes and enzymatic activities present in one kingdom that are not available in another (Lapierre and Gogarten, 2009; Lynch *et al*., 2011). Overcoming these limitations has been challenging using intrakingdom strategies. For example, previous studies have found it difficult to express eukaryotic enzymes in bacteria (Chang *et al*., 2007), while high‐yield production of certain compounds has also been difficult to obtain in engineered yeasts (Ajikumar *et al*., 2010).

Through the utilization of interkingdom co‐cultures, phylogenetically distant species can be gathered to gain novel functionality and higher production efficiency (Muñoz and Guieysse, 2006; Lindemann *et al*., 2016). Several disciplines and fields can benefit from these consortia, including environmental engineering, biosynthesis of fuel and production of commodity chemicals (Bernstein and Carlson, 2012; Minty *et al*., 2013). For instance, novel functionality was achieved within a bacterial–fungal co‐culture to create special flavours during fermentation in the food industry (Scherlach *et al*., 2013), and the addition of bacteria to an algal culture led to improved algal growth and enhanced biofuel production (Bagwell *et al*., 2014).

Independent of bioengineering practices, interkingdom microbial consortia are naturally found in diverse environmental compartments (further described as natural interkingdom consortia in this review), including extreme environments (e.g. hot springs, seabeds and subglacial melt), constituting a way of life with more effective and efficient growth than monopopulations (Paerl and Pinckney, 1996; Warren and Kauffman, 2003; Jorgensen *et al*., 2012). These natural interkingdom consortia play a critical role in ecosystems and global processes that spans biochemical cycling of elements (e.g. carbon, nitrogen and sulfur), geochemical reactions (e.g. mineral precipitation and dissolution) and climate change (Warren and Kauffman, 2003; Jorgensen *et al*., 2012; Prokopenko *et al*., 2013; Wagner, 2015; Wegener *et al*., 2015; Dang and Lovell, 2016; Zhang *et al*., 2017). Furthermore, interkingdom consortia have been widely found and extensively studied in the human body, such as biofilms in the oral cavity and human gut commensal microbiota (Thakur *et al*., 2016; Zuñiga *et al*., 2017).

Important interactions, mechanisms and structures determined in studies of natural interkingdom consortia are critical first steps in the design of an artificial interkingdom consortium. For example, in natural consortia found within the human oral microbiome, the bacteria *Streptococcus mutans* cooperates with the yeast *Candida albicans*, enabling the formation of a strong biofilm on teeth (Hwang *et al*., 2017). While this negatively impacts the human host and causes challenges for oral treatment options, this bacterial–fungal relationship may facilitate other applications requiring strong biofilm formation. Indeed, adhesion strength may lead to higher power densities in microbial fuel cells because this would enable a larger number of bacteria to contribute to power generation (Logan, 2009).

**Figure 1:**
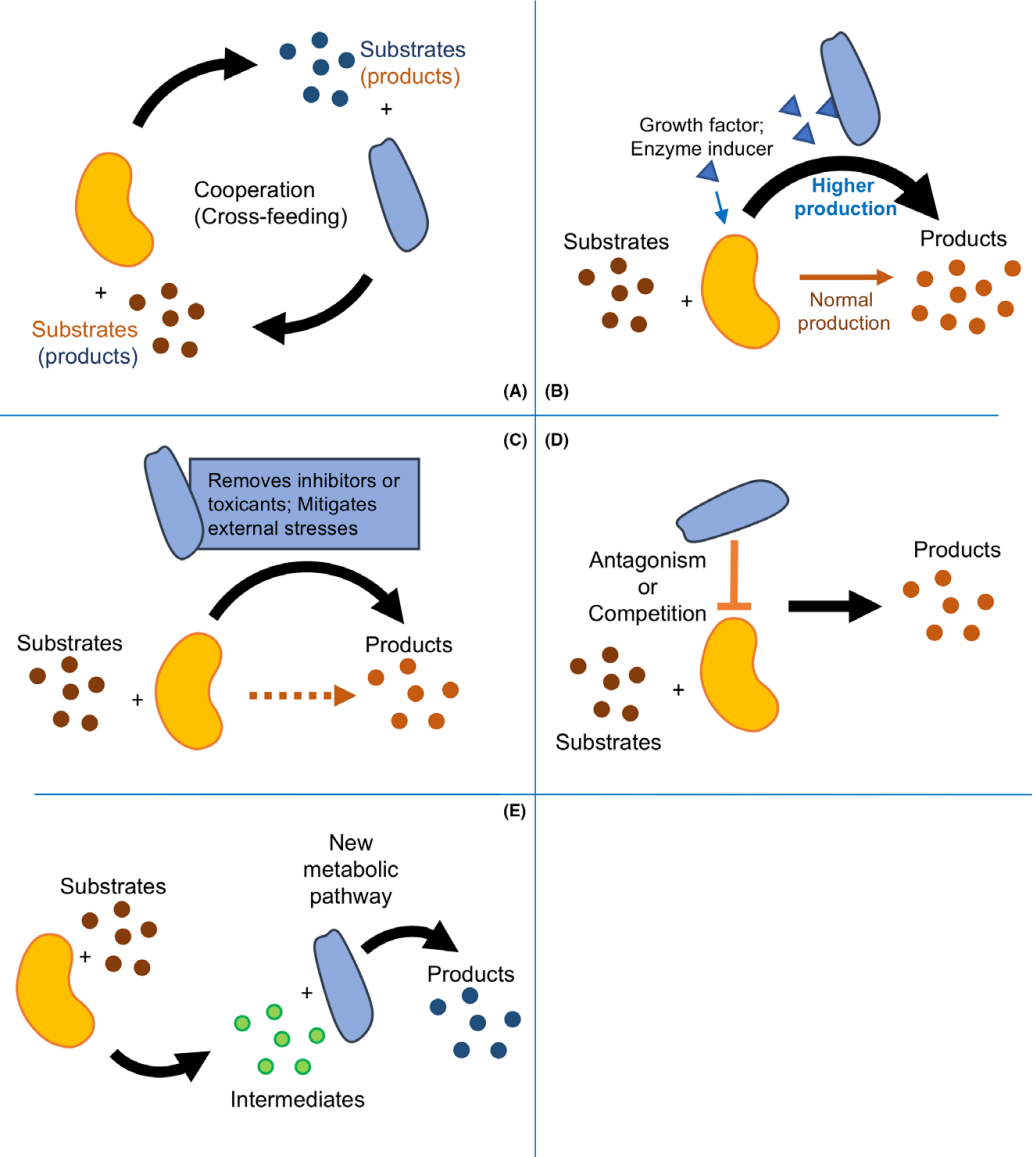
Schematic illustration of microbial consortia mechanisms. Cells represent microbial species from different kingdoms, including archaea, bacteria, fungi and algae. A. Synergistic division of resources (e.g. cross‐feeding) and expansion of the resources spectrum (beneficial interactions). In general, cooperation within the microbial consortia can improve microbial metabolism, as considered in division of labour. B. Stimulated microbial growth and biotransformation. This commensal relationship is a type of symbiosis. C. Enhanced tolerance of inhibitors or toxicants to mitigate external stress and inhibition. This relationship can be achieved *via* inhibitor or toxicant removal by partner species. The dotted arrow represents decreased or inhibited product formation. D. Antagonistic interactions lead to production of beneficial metabolites and enzymes that may not be produced otherwise. 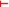 represents antagonistic or competitive interactions. E. Assembled biotransformation pathway to optimize efficiency and improve consortia robustness.

**Figure 2:**
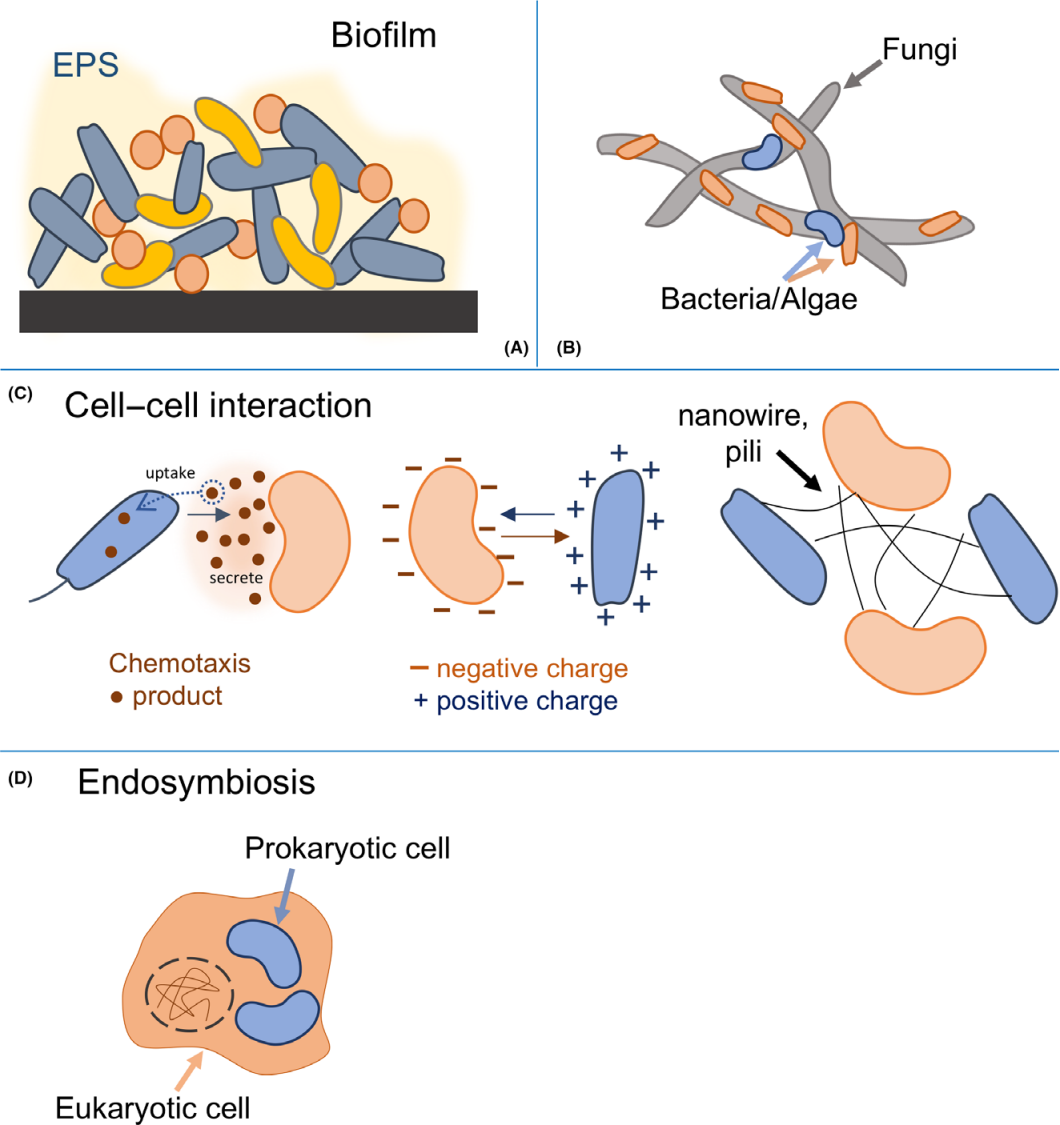
Schematic depiction of spatial interactions in microbial consortia. A. Biofilms are matrix‐enclosed microbial populations co‐localized to surfaces or interfaces and have been applied for bioprocessing and biotechnological purposes via artificial design. B. Symbiotic interaction among diverse cells including filamentous species (e.g. fungi) and other microorganisms (e.g. bacteria and algae) by surface attachment. C. Cell–cell interaction, including chemotaxis response induced by small diffusible molecules secreted by microorganisms; interactions related to positive and negative surface charges and attachment of nanowire/nanotubes between species to transport growth essentials and communication signals, such as electrons and protons. D. Endosymbiosis consists of one or more prokaryotic species living within a host cell.

This review presents our current understanding of the ecological principles (e.g. interactions, regulations and functionalities) of interkingdom consortia (Figs 1 and 2; Tables 1 and 2) and proposes parameters which can be controlled (e.g. metabolite and enzyme production) to manipulate consortia outcomes for beneficial applications. These novel insights will uncover common and unique features of interkingdom consortia compared to those in intra‐kingdom ones, further providing guidance in the construction of artificial microbial consortia for biotechnological applications. In addition to ecological principles, this review will also include recent technological developments that can enhance our understanding of interkingdom relationships and provide the means to discover unknown interactions and functions.

**Table 1:** Materials that influence interkingdom microbial consortia mechanisms and examples

Materials exchanged	Purpose	Examples			
		Microorganism(s) 1	Direction of exchange	Microorganism(s) 2	Ref
Nutrients (carbon, nitrogen, amino acids, sugar)	Growth support	Bacteria	CO_2_ → ← O_2_	Algae	(Muñoz *et al*., 2006)
Trace elements	Growth support	*Vibrionaceae* (marine anaerobe)	→ Iron	Other biofilm members	(Payne *et al*., 2016)
Vitamins	Growth support	Mixed‐species bacterial biofilm	→ Vit B_12_ a	*Chlorella vulgaris* and *Scenedesmus obliquus* (microalgae)	(Krohn‐Molt *et al*., 2013)
Phytohormones	Growth support to cell division and differentiation	*Azospirillum brasilense* (bacterium)	→ indole‐3‐acetic acid (IAA)	*Chlorella vulgaris* Beij. (microalgae)	(De‐Bashan *et al*., 2008)
Chelators/siderophores	Concentrate trace metal ions Increase nutrient bioavailability Solubilize metals	Marine bacteria	→ Siderophore enhances iron uptake	Phytoplankton	(Amin *et al*., 2012)
Proteins/enzymes	Interspecies predation Social behaviour	Parviluciferasinerae (parasitoid)	← dimethylsulphide (DMS) activates *P. Sinerae* from dormant stage	*Alexandrium minutum* (dinoflagellate)	(Garcés *et al*., 2013; Alcolombri *et al*., 2015)
Electrons (e‐, hydrogen, formate)	Improved growth	Archaea (anaerobic methane oxidizing)	⟷ electrons	Sulfate‐reducing bacteria	(Wegener *et al*., 2015)
Secondary metabolites	Antibiotics, stress response	*Burkholderia* sp. (rhizoxin‐producing bacterium) *Aspergillus fumigatus* (airborne fungi)	← Protection ⟷ Nutrients from decaying plant due to rhizoxin ← Production of silent fungal secondary metabolites	*Rhizopus* sp. (fungus) *Streptomyces peucetius* (bacterium)	(Partida‐Martinez and Hertweck, 2005; Luti and Mavituna, 2011; Marmann *et al*., 2014)
Genes (plasmid, phage)	Prokaryotic speciation	Phage	→ Horizontal gene transfer	*Chlorobiaceae* sp. (green sulfur bacterium)	(Llorens‐Mares *et al*., 2017)
Signals (quorum sensing)	Cell–cell communication Regulate microbial processes (extracellular enzyme production, population density, antibiotic production and biofilm formation)	Diatoms *E. coli*	→ Pheromones → Volatile acetaldehyde	Bacteria in consortia can attach to extracellular organic biomolecules Mammalian cell (CHO‐sBLA expresses BLA, triggering ampicillin degrader production)	(Weber *et al*., 2007; Amin *et al*., 2012)
Extracellular polymeric substances (EPSs)	Protection Electron/signal channel Enhanced environmental stability	Diatoms (support attach growth and carbon source) *Salmonella enterica*(bacterium)	→ Community ‘building blocks’ and carbohydrates ← Formation of hyphae on maize for bacterial biofilm construction	Bacteria *Aspergillus niger* (fungus)	(Taylor *et al*., 2013; Battin *et al*., 2016; van Overbeek and Saikkonen, 2016)

**a**. Other interactions could be found between the bacterial biofilm and algal species, such as the production of EPS. EPS consists of mostly fatty acids produced from the microalgae and also contains polysaccharides, proteins, nucleic acids and lipids. These EPS constituents provide stability, structure and defensive mechanisms.

**Table 2:** Important interkingdom consortia features with an example(s) demonstrating biotechnological application

Main consortia feature	Microorganisms in the interkingdom consortia	Starting compound ⇉ Product	Output amount (time)	Ref
Modularity—combining high‐yield production (e.g. bacteria) with functionalization (e.g. fungi)	*Escherichia coli* (bacteria) /*Saccharomyces cerevisiae* (fungi)	D‐xylose ⇉naringenin	21.16 mg l^−1^ (96 h)	(Zhang *et al*., 2017)
	*Escherichia coli* (bacteria) /*Saccharomyces cerevisiae* (fungi)	Taxadiene⇉ oxygenated taxanes	33 mg l^−1^ (120 h)	(Zhou *et al*., 2015)
Modularity—various end products produced	*Escherichia coli* (bacteria) /*Saccharomyces cerevisiae* (fungi)	Dopamine ⇉ various benzylisoquinoline alkaloids	55 mg l^−1^ (*S*)‐reticuline (1 h)	(Minami *et al*., 2008)
			7.2 mg l^−1^ magnoflorine (72 h)	
			8.3 mg l^−1^ scoulerine (48 h)	
Expansion of the growth substrate spectrum	*Actinotaleafermentans* (bacteria)*/Saccharomyces cerevisiae* (fungi)	Cellulose feedstocks⇉ acetate/ethanol ⇉ methyl iodide	~20–150 mg l^−1^‐h (36 h)	(Bayer *et al*., 2009)
	*Saccharomyces cerevisiae* (fungi)/*Shewanella oneidensis* (bacteria)	Glucose (or other carbon sources) ⇉ lactic acid ⇉ bioelectricity	123.4 mW m^−2^	(Lin *et al*., 2017)
Cooperator–cheater stability	*Trichoderma reesei* (fungi)*/Escherichia coli* (bacteria)	Cellulose and corn stover⇉ isobutanol	1.88 g l^−1^ (~400 h)	(Minty *et al*., 2013)
Cell–cell adhesion (bioflocculation, physical interaction)	*Aspergillus fumigatus* (fungi)/algae (11 strains)	Various waste sources ⇉ wastewater treatment and lipid production	~77–87% removal ${\text{NH}}_{4}^{-}$ (48 h)	(Wrede *et al*., 2014)
			~50–68% removal ${\text{PO}}_{4}^{3-}$ (48 h)	
			~18–246 mg l^−1^ lipids (48 h)	
Production of novel metabolites through interkingdom consortia design	*Fusarium tricinctum* (fungi)/*Bacillus subtilis* 168 trpC2 (bacteria)	Solid rice medium ⇉ secondary metabolites	(3 weeks growth) ˜0.7 mg macrocarpon C ˜4 mg (–)‐citreoisocoumarin ˜16 mg 2‐(carboxymethylamino)benzoic acid ˜0.4 mg (–)‐citreoisocoumarinol ˜4.9 mg lateropyrone (antibacterial) ˜22–89 mg Three cyclic depsipeptides (enniatin type) (two were antibacterial) ˜79 mg lipopeptide fusaristatin A	(Ola *et al*., 2013; Marmann *et al*., 2014; Marmann *et al*., 2014; Netzker *et al*., 2015)
Endosymbiotic interactions promote desired metabolite production	*Rhizopus microsporus* (fungi)/*Burkholderia* spp. (bacteria)	Fermentation medium ⇉rhizoxin (biosynthesis is lost in pure cultures)	Not available	(Partida‐Martinez and Hertweck, 2005; Schmitt *et al*., 2008)

## Cooperation within interkingdom consortia broadens the substrate spectrum

Cooperation, or syntrophy, is one of the fundamental principles followed by natural and artificial microbial consortia to maintain a long‐term stable lifestyle (Morris *et al*., 2013; Spribille *et al*., 2016) To overcome resource scarcity in nature, microorganisms create a ‘division of labour’ or ‘resource division network’ to support individual members, including the production, transfer and utilization of resources (e.g. electron donors and acceptors, trace metals and carbon sources) (Fig. 1A) (Bernstein and Carlson, 2012; Payne *et al*., 2016). One major avenue for microorganisms to participate in a ‘resource division network’ is cross‐feeding, in which the metabolites produced by one member are utilized by another (Fig. 1A) (Morris *et al*., 2013), either through passive or through active uptake strategies (e.g. nanotubes, clumping, diffusion and membrane transport proteins) (Fig. 2). The exchange of diffusible metabolites (e.g. hydrogen, formate, methanethiol, amino acids or carbohydrates) and communication signals between consortia members can improve the end goal of an artificial consortia (e.g. high‐yield production) compared to monocultures and can significantly broaden the carbon and energy spectrum for each member (Fig. 1B). Metabolite exchange is common in natural consortia and may also result in cooperative cross‐feeding, in which a member of the consortia has evolved to begin producing increasing amounts of a certain metabolite for the benefit of others (Pande and Kost, 2017). This mechanism has been repeatedly exploited in interkingdom biotechnological applications to produce desirable products sustainably via sharing and dividing the production of metabolites, reducing the metabolic burden (Wu *et al*., 2016) and improving fitness and stability (Pande and Kost, 2017) for each microbial member (Tables 1 and 2).

Several challenges (e.g. the growth rate of each species and the production of inhibitory compounds) to constructing an artificial consortium can be mitigated by cooperative relationships (Fig. 1C). Mutualistic associations can be engineered to ensure that each species depends on each other for survival while enabling modularity and high‐yield synthesis of certain compounds, including complex natural compounds. A module system divides the metabolic pathway among each consortia member and reduces the metabolic burden, improving cell fitness and resulting in higher yields compared to monocultures (Zhang and Wang, 2016).

In simple, interkingdom consortia containing two species, Zhou and co‐workers (Zhou *et al*., 2015) demonstrated that *Saccharomyces cerevisiae* and *Escherichia coli* were able to maintain a stable relationship through several genetic changes for improved production of an antitumor agent. The consortia relied on a mutual relationship where xylose metabolism in *E. coli* released acetate as a by‐product that could be utilized by *S. cerevisiae* as the sole carbon source. *E. coli* are sensitive to acetate concentrations; therefore, this mutual relationship enabled increased growth of both bacterium and yeast. Furthermore, the modular design of this engineered consortia allowed individual and parallel optimization which resulted in better consortia performance (Zhou *et al*., 2015), where *E. coli* efficiently produced antitumor precursors and *S. cerevisiae* functionalized these precursors using more complex enzymatic processes. This study further demonstrates that interkingdom consortia can be utilized even for the synthesis of complex pharmaceutical and therapeutic compounds, such as alkaloids and flavonoids. Moreover, depending on the interkingdom consortia designed, various carbon sources could be used to produce the desired effects, enabling flexibility (Table 2) (Lin *et al*., 2017). Further tests for each consortium should also be carried out to ensure the optimum ratio of each species (starting inoculum), starting compound and high‐yield production. Modelling, especially flux balance analysis (FBA) (Orth *et al*., 2010; Wu *et al*., 2016), can help predict the starting parameters for experimentation and further optimization to promote a cooperative relationship [e.g. Computation of Microbial Ecosystems in Time and Space (COMETS) (Harcombe *et al*., 2014) or a simple phenomenological model (Hoek *et al*., 2016)].

However, a challenge to overcome in the cooperative consortia is the presence of ‘cheaters’. Cheaters utilize resources and products without participating in maintaining community fitness and survivability (Kümmerli *et al*., 2015). Cheaters in biotechnological applications are not limited to within species dynamics but can extend to interkingdom, cooperative relationships ‘cooperator–cheater dynamic’ (Kümmerli *et al*., 2015) depending on the construction of the artificial consortia. Many natural microbial consortia also have communication signals and cues (Stacy *et al*., 2012) that could be exploited in artificial consortia to inhibit the occurrence of cheaters. Recent applications of game theory modelling have helped to prevent cheaters in long‐term artificial consortia (Schuster *et al*., 2010; Minty *et al*., 2013).

The cheater population is especially important to track when designing evolution or genetic experiments for biotechnology due to the high potential for a cheater population to emerge. One possibility for tracking cheaters is through the use of a ‘negative and positive selection scheme’, which has yet to be applied to multispecies consortia. This method was developed by Raman *et al*. (2014) when inducing for mutations in *E. coli* to obtain highly efficient producers of the target compounds (naringenin or glucaric acid) through several rounds of evolution. Unfortunately, this also led to the evolution of some *E. coli* cells which were utilizing resources, but did not produce the target compound (cheaters). A biosensor was used to track the production of the target compounds, while a selector (TolC) was used for the selection of efficient producers. Through each round of evolution, cheaters were removed from the *E. coli* culture, while the most efficient producers were captured. If this concept is applied to multispecies consortia, it has the potential to prevent cooperator–cheater dynamics while also selecting for the optimal consortia composition.

## Interkingdom consortia have enhanced tolerance towards external stress and inhibition

In general, microbial consortia display adaptive strategies to survive extreme environments and disruptive events. Intrakingdom consortia maintain stability and strengthen defences against external stresses by physical and spatial structure modifications (Smith *et al*., 2015; Flemming *et al*., 2016) (Fig. 2). These are also present in interkingdom consortia (Agapakis *et al*., 2012; Stal, 2012) but with more variety, complexity and possibilities for interaction (Flemming and Wingender, 2010; Scherlach *et al*., 2013; Kean *et al*., 2017). Consortia can physically interact via aggregation (e.g. chemotactic responses) (Seymour *et al*., 2010; Sourjik and Wingreen, 2012), surface charge (e.g. fungal–algal communities) (Wan *et al*., 2015) or immobilization (e.g. filamentous microorganisms acting as sorbents and sources of nutrients for the surface attachment of others) (Hogan and Kolter, 2002). Natural microbial mats, which can be composed of interkingdom species (Riding, 2000), are an excellent model to demonstrate the metabolic complementarity of a microbial consortia, including the distribution of oxygen, light and redox gradients (Stal, 2012). Some members within this compartmentalization can also remove undesirable metabolites and reduce competing reactions (Kim *et al*., 2015; Bernstein *et al*., 2017), thereby mitigating any negative impacts posed by inhibitors or toxicants towards a critical population (Fig. 1C). This becomes an important advantage for interkingdom microbial consortia applications [e.g. biofuel production and bioenergy conversion (Nishio *et al*., 2010; Wang *et al*., 2012; Agbakpe *et al*., 2014)], which utilize physical and spatial organization.

Interkingdom artificial consortia design can also capitalize on the production of extracellular polymeric substances (EPSs) (Fig. 2A). EPS is commonly produced by bacterial, fungal and algal cells and enables resistance to environmental stresses, potential toxins and phage attacks (Costerton *et al*., 1995; Sutherland, 2001; Wingender *et al*., 2012). Both in the biofilm and planktonic microenvironment, EPS functions as a biopolymer barrier for the community and can be an alternative energy resource under nutrient‐depleted conditions (Zhang and Bishop, 2003). For instance, *Fomitopsis pinicola* produces EPS to scavenge hydroxyl radicals and to block UV damage, thus maintaining the viability and activity of the surrounding microorganisms (Hao *et al*., 2016). EPS can also help partition an anaerobic microenvironment by preventing contact between anoxic species and oxygen (Elliott *et al*., 2014), further demonstrating the applicability of EPS for artificial consortia. As EPS has the potential to provide a protective shield and prevent growth essentials diffusing outward (Mueller and González, 2014), construction of an artificial consortia with EPS‐producing members and other interkingdom species would further assist in consortia maintenance, resistance and tolerance of inhibitory elements.

Other interactions [e.g. symbiosis, formation of extracellular structures and transfer of mobile genetic elements (MGE)] can occur in a consortium's network that further strengthens each consortium member against external stresses (Fig. 2B). For example, it was determined that symbiotic relationships within a diverse interkingdom consortia consisting of plants, bacteria and fungi played an important role as mediators of nutrient supply in nutrient poor soils (van der Heijden *et al*., 2015). Numerous studies have shown that MGE (e.g. plasmids, transposons, integrons, genomic islands or phage) and horizontal gene transfer (HGT) events occur across kingdoms. A previous study by Marcet‐Houben and Gabaldón (2010) analysed and sequenced 60 fungal genomes and identified 713 prokaryotic genes likely transferred via HGT events. The integration of MGE and HGT provides a means to regulate and control certain functions present in the consortia by directly introducing microorganisms containing specific genes, such as genes involved in bioremediation (Top *et al*., 2002). This approach becomes increasingly beneficial if the recipient of genetic material is suited better to the environmental conditions. Gene transfer may also occur via microbial extracellular structures, such as nanowires and pili (Fig. 2C) (Flemming and Wingender, 2010). Nanowires have recently been shown to occur between bacteria and archaea (Wegener *et al*., 2015), and while interkingdom nanowires have yet to play a large role in biotechnology applications, intra‐kingdom nanowires already promote direct electron transfer for electromicrobiology applications (Nealson and Rowe, 2016) (e.g. microbial fuel cells and wastewater treatment plants).

Future interkingdom microbial consortia design can also exploit the CRISPR‐Cas systems, a microbial strategy to overcome ‘invasions’ by phages/viruses. While recent studies utilize CRISPR in monoculture systems (Donohoue *et al*., 2017), the new discovery of the signalling pathway between Cas and Csm complexes uncovered a future research direction of CRISPR regulation by controlling the signal messengers that activate the RNase activity (Kazlauskiene *et al*., 2017; Niewoehner *et al*., 2017). Although the CRISPR evolution and adaptation at the consortia level are still limited (Davison *et al*., 2016; Burstein *et al*., 2017), the manipulation of communication signals may be feasible in microbial consortia and further adapted to other biotechnological applications.

## Antagonistic interactions within interkingdom consortia can lead to the production of beneficial metabolites and enzymes

Competitive or antagonistic interactions that partake in microbial consortia generally arise during resource and space limitations, potentially resulting in the production of secondary metabolites (Table 1) and the activation of silent genes (Fig. 1D). Competition can be split into two categories: interference competition to negatively impact other species by producing harmful products and exploitative competition to compete for scarce resources. These competitive interactions can be an attractive factor in the biotechnological applications of interkingdom consortia with the potential to improve product yields and further expand the possible metabolites produced when co‐culturing diverse microorganisms (Hailei *et al*., 2013; Marmann *et al*., 2014). These interactions are also known to promote fitness, diversification and niche adaptations as each species evolves to survive within the community (Watanabe *et al*., 1982; Michie *et al*., 2016).

Synthesis of novel compounds or secondary metabolites can be provoked by interspecies competition due to the activation of genes that would otherwise remain silent in monoculture systems (Watanabe *et al*., 1982). Generally, competitive interactions occur when species occupy the same niche, resulting in the evolution of certain species to produce signals that can detect potential competitors (Michie *et al*., 2016). Once detected, microorganisms can produce substances that cause oxidative stress, DNA and/or cell wall damage, which can be negated by other species through the biosynthesis of novel compounds or secondary metabolites such as antimicrobial compounds and other self‐defence mechanisms. In particular, antimicrobials have evolved to target multiple species or phylogenetically similar strains (Mitri and Foster, 2013), and current research into new antimicrobials has taken advantage of interkingdom consortia. For example, microbial consortia containing fungi and bacteria have been used to obtain new antibiotics, such as aspergicin and lateritin (Marmann *et al*., 2014; Netzker *et al*., 2015).

Certain enzymes [e.g. extracellular oxidoreductases (Flores *et al*., 2009)] may also only be produced by mixed consortia due to competitive interactions resulting in microbial stress. While these enzymes could be promoted by the introduction of abiotic inducers, including aromatic compounds, copper and ethanol, these inducers may be toxic or cost prohibitive for practical applications. To the authors’ knowledge, enzyme production by abiotic inducers has only been demonstrated in intra‐kingdom consortia. Specifically, co‐cultivation of wood‐decaying fungi (Basidiomycetes) with filamentous fungi promoted the production of extracellular oxidoreductases without an abiotic inducer (Flores *et al*., 2009). Generally, these extracellular oxidoreductases are the result of an accelerated switch to secondary metabolism due to oxidative stress and competition for nutrients and space. Thus, by exploiting the competitive fungal mechanisms, these extracellular oxidoreductases can then be used for biofuels, paper–pulp industry, bioremediation and agricultural remediation (Bertrand *et al*., 2014). This intra‐kingdom example demonstrates the applicability of antagonistic interactions that could arise within interkingdom consortia. The production of certain enzymes, compounds or metabolites may only be derived from these competitive mechanisms, and future studies are needed to identify the fundamental pathways to parse out applicable microorganisms that can be maintained for cost‐effective, interkingdom consortia design.

## Interkingdom consortia exhibit novel metabolic pathways for yielding chemicals

Interkingdom microbial consortia operate as a type of ‘chemical reactor’ to achieve cost‐effective metabolic outputs for chemical engineers (Dueber *et al*., 2009). As described previously, module consortia design generally helps to achieve desired outputs while providing flexibility for optimization strategies and nutrient requirements (e.g. carbon sources), but using interkingdom consortia design heterologous microbial functions can be utilized to assemble novel biochemical pathways and generate unique products (Fig. 1E). For example, volatile organic compounds produced by the fungus *Fusarium culmorum* induced the expression of a wide range of genes and proteins from the bacterium *Serratia plymuthica* including the production of a previously unknown terpene sodorifen that may play a role in fungal–bacterial long‐distance communication (Schmidt *et al*., 2017). In consortia containing fungi and bacteria (or algae), fungi often act as ‘dispersion vectors’ for others, increasing nutrient availability and providing physical protection. These dispersion vectors can help to achieve the desired results, including improved biotransformation of polycyclic aromatic hydrocarbons (Zhang *et al*., 2014; van Overbeek and Saikkonen, 2016), which may not be achievable with monoculture or intrakingdom design.

Further demonstration of achieving novel functionality in interkingdom design includes the construction of artificial endosymbiotic relationships, for example, to gain the function of chloroplasts in other host cells (Agapakis *et al*., 2011). In nature, the thermophilic, acidophile red algae (Cyanidiales) contain a plasmid with proteobacterial origins, arising from endosymbiotic behaviour (Fig. 2D) (Ciniglia *et al*., 2004). In general, multispecies consortia that eventually become one individual (e.g. red and green algae) can then participate in secondary and tertiary endosymbiotic relationships (e.g. heterokont algae, dinoflagellates and cryptophytes) (Pulz and Gross, 2004). These relationships can be exploited for biotechnological applications and to improve production of useful compounds, such as the development of synthetic microbial endosymbionts by manipulating plastids (e.g. a photosynthetic plastid) into a host cell (e.g. yeast) (Weber and Osteryoung, 2010).

Naturally occurring endosymbiotic consortia (e.g. between eukaryotic and bacterial partners) have also been used to produce useful biological compounds, such as rhizoxin, which has potential use as an anticancer agent (Partida‐Martinez and Hertweck, 2005). The rhizoxin‐producing bacterial–fungal partnership also has important implications for agriculture, biotechnology and fermented food production and was discovered by conducting stable isotope labelling studies and confirming with microscopy, molecular biology and bioinformatics. These methodologies further demonstrated the evolution of a parasitic–mutualistic shift in rhizoxin‐insensitive fungi over time (Partida‐Martinez and Hertweck, 2005; Schmitt *et al*., 2008). A combination of such tools—stable isotope analysis, microscopy and molecular biological techniques—can be utilized to reveal the microbial consortia physiology and the source and requirements for the biosynthesis of novel natural compounds.

## Potential methods to control the interkingdom interactions

Interactions within the interkingdom microbial communities are heterogeneous, complex and quite challenging to be deciphered, requiring additional studies to control, manipulate and optimize these consortia for beneficial applications. Numerous techniques and devices developed for intrakingdom analyses may be applicable for interkingdom studies and provide the appropriate tools to investigate the control of function and efficiency of interkingdom reactions.

### Identification of microbial members for interkingdom consortia construction

Prior to biotechnological and industrial applications, members of a consortium must be isolated, either in mono‐ or in co‐culture, to allow for further studies. High‐throughput isolation and enrichment of natural microbial members can be obtained by such devices as the iChip (Nichols *et al*., 2010). These devices enable the isolation of uncultivable microorganisms compared to traditional methods (e.g. plate streaking). For example, the iChip is a diffusion device which allows for simultaneous *in situ* incubation of hundreds of individual cells. Although physical contact is not possible for cells growing in the iChip, metabolites can be shared between all isolates (e.g. signalling molecules, growth factors, enzyme inducers), making it possible to identify a wide variety of microorganisms. After isolation of consortia members, it is possible to conduct targeted experiments and genome sequencing to reveal the possible consortia compositions that are responsible for the production of specific metabolites [e.g. antimicrobials (Ling *et al*., 2015)], functions and interactions (Lindemann *et al*., 2016).

Future studies are also required to understand the effects of competition over long‐term application of microbial consortia and to optimize the biotechnological potential of competing microorganisms. For example, when selecting microorganisms to partake in competitive interactions for biotechnological applications, long‐term maintenance of the consortia structure could be affected by the synthesis of unwanted defensive products [e.g. diatoms produce fatty acids and esters that impact algicidal bacteria and affect the bacterial composition in the consortia (Amin *et al*., 2012)]. By expanding this strategy to other systems (e.g. biofuel production, microbial fuel cells, biological water treatment processes) and other members of a microbial consortium, there is potential to reveal rate‐limiting roles of specific community members in important biotransformation processes.

### Metabolites identification

The most direct benefit from artificial microbial consortia is the products, either newly synthesized or overexpressed. Metabolites produced from natural or artificial interkingdom communities can be achieved using high‐performance liquid chromatography (HPLC), liquid chromatography‐tandem mass spectrometry (LC‐MS/MS) or nuclear magnetic resonance spectroscopy (NMR) (Caraballo‐Rodríguez *et al*., 2017). Moreover, mass spectrometry imaging (MSI) is an emerging technique, including secondary ion mass spectrometry (SIMS) (Sheik *et al*., 2016; Paine *et al*., 2017) and Raman scattering microspectroscopy (Bodelon *et al*., 2016; Wang *et al*., 2016), which enables the visualization of metabolites in complex samples, such as environmental mixtures and microbial consortia. These advanced analytical techniques can assist the identification of biological products along the metabolic pathway, providing references to improve production efficiency and to develop novel biological products.

### Biosensors and communication signals

Biological recognition elements may serve as biosensors and universal signalling molecules, which can be manipulated to regulate microbial activities and to provide (semi‐) quantitative analytical information, such as in bacterial/fungal interactions, the human oral microbiome and the food industry (Kolenbrander *et al*., 2010; Bertels *et al*., 2012; Gao *et al*., 2016; Manoharan *et al*., 2018). Biosensors, including enzymes, antibodies, whole cells and ligands, can be used without analytical instruments (e.g. gas and liquid chromatography) and have been used to measure metals, dissolved gases, substrates, proteins and more (Thévenot *et al*., 2001). Through the use of biosensors, cost‐efficient analysis of reactions occurring in an interkingdom system is possible, providing ample opportunity for manipulation and control. Interkingdom biosensors that include bacteria and fungi have displayed higher sensitivity and broader chemical sensing functions than single species devices (Gao *et al*., 2016). Because of these interactions and communication signals among species, designable functions can be achieved by regulating or engineering consortia composition, growth factors and gene regulation (Agapakis *et al*., 2012). For example, accelerated fermentation and methane production was observed in a bacterial and archaeal microbial consortia due to enhanced direct interspecies electron transfer (Li *et al*., 2015). Emerging studies on the transportation of small RNA between fungi and plants (Zhou *et al*., 2017) and on the nucleotide transport proteins among bacteria and eukaryotes (Major *et al*., 2017) also reveal the versatile communication strategies in a microbial consortium. These findings will provide opportunities to utilize communication traits to control interkingdom consortia for advanced applications.

Communication strategies are not limited to prokaryotic and eukaryotic cells but can also be applied to viruses. Viruses have the potential to increase a host's fitness and can become mutualistic partners with some bacteria (Weinbauer, 2004). For example, viruses contain many genes related to virulence, stress response and motility (Weinbauer, 2004) and several bacterial genomes contain viral genes, including pathogenic and photosynthetic bacteria. This resulted from viruses acting as a vector for genes, leading to evolutionary microbial diversification and increased fitness (Weinbauer, 2004; Moran, 2007). Currently, viruses have been applied to a consortium for their negative impact on microorganisms, such as the utilization of viruses for antimicrobial therapy to degrade bacterial biofilms (Lu and Collins, 2007). However, viral infection could also be used as a means to regulate the host's population via infection and could help maintain long‐term, engineered microbial consortia. Therefore, efforts focusing on viral infections within interkingdom microbial communities are still required.

### Meta‐omic analyses

The utilization of more advanced microbial metabolic and molecular biological techniques, including metagenomics, metatranscriptomics and metabolomics, can elucidate the possible secondary metabolites microorganisms can produce and focus on microbial consortia design towards the activation of silent genes. For instance, metatranscriptomic analyses have demonstrated that fungal mixed cultures can change the production of monosaccharides and secondary metabolites, in addition to the gene expression levels of lignocellulolytic enzymes, oxidoreductases and cell wall hydrolases (Daly *et al*., 2017). Furthermore, by conducting metabolomics on *Aspergillus fumigatus*, at least 226 secondary metabolites, including silent gene clusters, were discovered. Co‐cultivation with bacteria such as *Streptomyces peucetius* and *Streptomyces bullii* could induce these silent genes in *A. fumigatus*, enabling the production of new metabolites with biotechnological applications (Netzker *et al*., 2015). Advanced meta‐omic analyses have furthered our understanding of these interactions and the role of the consortia and community structure for important biotransformation processes (Zuñiga *et al*., 2017). However, there are potential pitfalls in meta‐omic analyses among various kingdoms due to the nature and method of analysis; for example, amplicon sequencing of portions in the 16S rRNA region (bacteria) and the ITS and 18S rRNA region (eukaryotes) limits the identification of microorganisms (e.g. primer bias) and potentially skews the resulting microbial diversity and abundances. Data interpretation is often under the assumption of the identified consortia or community. As technology continues to advance (e.g. full‐length 16S rRNA retrieval (Singer *et al*., 2016) and full‐length ITS and 18S rRNA regions) and as research utilizes concepts and tools from various fields, further insights into the interkingdom microbial mechanisms can expand our knowledge, improve current microbial applications and promote new research directions.

## Concluding remarks

Interkingdom microbial interactions between archaea, bacteria, fungi and algae provide societal benefits in natural and engineered systems that are unlikely to be achieved in monocultures or intrakingdom consortia due to a lack of promoting symbiotic or competitive functions. Biotechnological advances allow for the development of consortia from different natural environments without the need for genetic engineering or optimization of nutrients and cell ratios (Hom and Murray, 2014). Similarly, emerging analytical tools targeting specific metabolites and genes can be used to manipulate synthetic microbial consortia by taking advantage of the microbial mechanisms discussed in this review. Further identification of new microorganisms, special microbial communication patterns and novel metabolites will promote interkingdom consortia construction for cost‐effective design and enhanced microbial applications, including bioremediation, bioenergy and biomedical. Importantly, understanding microbial communication and interactions within interkingdom consortia will provide references to study interactions between microorganisms and higher‐level species (Oh *et al*., 2014), such as humans, animals and plants, and could even extend to their pathogenicity and/or immune defences.

## Conflict of interest

The authors declare there are no potential conflicts of interest regarding this review.

## Definitions

*Microbial consortia* are described as a microbial association containing at least two or more microbial members with certain lifestyle(s) (Paerl and Pinckney, 1996). Synonyms include ‘hybrid consortia’, ‘multispecies consortia’, ‘mixed cultures’, ‘mixed consortia’ and ‘co‐culture’ (Brune and Bayer, 2012; Großkopf and Soyer, 2014; Song *et al*., 2014; Lindemann *et al*., 2016). The microorganisms found within a consortium include not only bacteria but also archaea, fungi, viruses and algae.

*Microbial community* consists of ‘multispecies assemblages/consortia, in which organisms live together in a contiguous environment and interact with each other’ (Konopka, 2009), although this definition is not comprehensive (Zarraonaindia *et al*., 2013).

*Artificial microbial consortia* include both engineered and synthetic microbial consortia.

*Engineered microbial consortia* can be defined as containing at least one genetically modified microbial member.

*Synthetic microbial consortia* consist of selected microorganisms which play different roles within the consortia (Großkopf and Soyer, 2014; Song *et al*., 2014).

*Endosymbiosis* is also a unique relationship and consists of one species living inside another for further protection and direct uptake of substrates and nutrients for growth.
